# Supplementary material for: Three heads are better than two: Comparing learning properties and performances across individuals, dyads, and triads through a computational approach
Source: PLoS One. 2021 Jun 17;16(6):e0252122. doi: 10.1371/journal.pone.0252122 (PMC8211165; doi:10.1371/journal.pone.0252122)
Supplement: S1 Appendix — (DOCX) [file pone.0252122.s001.docx]

**APPENDIX**

This appendix presents a simple model of the learning coherence of individuals and triads and the learning incoherence of dyads. Suppose that two learning strategies exist, $Sh$ and $Sl$, and that the ratio of the $Sh$ in the population is $p$. Strategies $Sh$ and $Sl$ yield expected rewards of $Rh$ and $Rl$ with $Rh>Rl$. However, other strategies, such as a randomized strategy between $Sh$ and $Sl$, are assumed to underperform compared to the previous strategies because they sacrifice learning coherence, thereby discouraging the utilization of cumulative learning. For sake of simplicity, it is assumed that the average expected rewards of such randomized strategies are $Rw$ with $Rw<Rl<Rh$. In dyads, when both members have different learning strategies, conflicts arise, leading to a situation in which both strategies are randomly adopted. The probability of adopting these strategies is $2p\left( 1-p \right).$On the other hand, in triads, it is expected that the majority decides which learning strategy to implement; that is, the coherent learning strategy is adopted without exception because, in triads, majority subgroups always exist.

The difference between the expected rewards for triads and dyads are $2p\left( 1-p \right)\left[ pRh+ \left( 1-p \right)Rl-Rw \right]>0$, indicating higher performance in triads. The difference in expected rewards between individuals and dyads are $p\left( 1-p \right)\left( Rh+Rl-2Rw \right)>0$. However, the relative performance of triads against individuals is $p\left( 1-p \right)\left( 2p-1 \right)\left( Rh+Rl \right)$, such that triads outperform individuals if $p>1/2$. Therefore, a U-shaped relationship in performance across individuals, dyads, and triads is probable; that is, individuals or triads perform relatively well (depending on $p$ in the population) and dyads perform relatively poorly due to the coherence and incoherence of their underlying learning strategies.
