# Supplementary material for: Three heads are better than two: Comparing learning properties and performances across individuals, dyads, and triads through a computational approach
Source: PLoS One. 2021 Jun 17;16(6):e0252122. doi: 10.1371/journal.pone.0252122 (PMC8211165; doi:10.1371/journal.pone.0252122)
Supplement: Attachment — Submitted filename: Revision List.docx [file pone.0252122.s002.docx]

**Revision List**

We greatly appreciate reviewers’ helpful comments and we believe we could significantly improve the quality of the paper. Here are how we revised the manuscript in response to the comments. The editor requested “It seems a major issue is to improve the modeling work and data analysis to where it is consistent with similar work in the reinforcement learning and mathematical modeling literature. You also need to better explain the key points and main goals for your study”. We believe by responding to reviewer’' requests and comments, these challenges were satisfied in the current revised manuscript.

Main changes
First, the main revisions in the current manuscript are as follows:

1. In response to the comment by Reviewer 1 that no theory was presented, we described a very simple model in Introduction that analyzed the performance differences across individuals, dyads, and triads, leading to the U-shaped relationship, which was caused by the coherence and incoherence of learning.
2. In response to Reviewer 2’s comment on the appropriateness of the Q learning model and suggestions to simplify the model and Reviewer 1’s inquiry into whether the results change or not when only one learning rate is introduced, we considered two models: (1) simple Q learning model with only one learning rate; (2) asymmetric learning model that only allowed for changing learning rates to the sign of reward prediction errors. The results in both models were the same: inverse temperature accounted for performance.
3. Following the advice by Reviewer 1, we examined within-group effects in which group learning parameters and the maximum, average and minimum of its individual group members’ learning parameters were compared.
4. Following the request by Reviewer 1, in analyzing group differences, we examined not only the pooled sample, but also the subsample in which all group members experienced both dyads and triads experiments. This subsample consists of 161 individuals, 56 dyads, and 42 triads. Although a few differences emerge between the pooled and subsample, main results remained the same.

Responses to Reviewer 1:

1. There is no theory. We do not know what to expect and WHY to expect it. … Moreover, the hypotheses and predictions should be fleshed-out and justified in the introduction. It is currently unclear what the hypotheses are.

- We introduced a simple model of learning of individuals, dyads, and triads, leading to the U-shaped relationship of group performance in the Introduction. This model follows the arguments of Simmel and proposition on even-sized groups vs. odd-sized group differences. We interpret these as learning coherence of odd-sized (individuals and triads in our manuscript) and learning incoherence of even-sized (dyads). The hypothesis was the U-shaped relationship across individuals, dyads, and triads, and learning inefficiency in dyads, which we believe were shown by our analysis.

2. However, very little is written about reinforcement learning in general, and Q-learning in particular. Relevant issues include the reasons for choosing to focus on learning (as opposed to perceptual decision-making in previous research), the ecological validity of this task, and what we already know about Q-learning in individuals.

- In Introduction, we added “Thus, this study adopted a reinforcement learning (RL) framework (34) to account for decision-making and learning behaviors in the two-armed bandit (TAB) problems, which is the standard model for model-based analysis of choice behavior. The RL framework has been extensively studied in the context of multi-armed bandit problems, in particular, with close association with neural signals in various cortical and subcortical structures that behaved as predicted (35-38). Moreover, the RL framework has also been adopted to study decision-making and learning in various social contexts (39-45). Nevertheless, to the best of our knowledge, this framework has not been applied to the study of group decision-making. One advantage of taking this computational approach is that learning parameters could be estimated as groups and also compared across and within groups of different sizes. “

3. There is no model comparison. … Do the results also hold in models with one learning rate, and without random noise? In a similar vein, do they also hold in a model that uses raw rewards, rather than prospect-utility-transformed values? Moreover, it can be illuminating to show potential differences in the best-fit model between the group sizes.

- In the previous model, we only considered only one variant of Q learning model. In this revised version, we considered (1) simple Q learning model with only one learning rate; (2) asymmetric learning model, and compared the model fit by calculating WBIC in the subsection “Model fits” in Results. The statistical test did not differentiate between the two models.

4. The design is unclear. … some participants did all three conditions but we do not know how many they were. Did any participants take part in more than 1 dyadic or triadic group? … it makes more sense if the dyadic condition is compared to individual performance of the subjects who took part in dyadic condition and exclude subjects who did not. A similar issue applies to triadic condition which should have its own individual control.

- Regarding the questions, we described “In test 2 (dyads), 23 participants played the TAB twice. In test 3 (triads), 72 participants played twice, and 14 participants played three times.” in Methods. In group comparison, we also considered the data in subsample in which all participants participated in dyads and triads, and compared the performance and learning parameters across three groups.

5. Also, if possible, it would be advisable to try out comparing triads to the best dyad pairing within a group. ...Such an analysis should not only be made on the level of overall performance, but also on the level of model parameters.

- Following this, we compared group performance and learning parameters and the max, min, and average of individual members’ performance and learning parameters. The results were described in the subsection Within group effects in Results.

6. Minor points

- “causes of synergy”　⇒ factors associated with synergy
- “To convert the group from…”: sentence unclear. ⇒ Deleted the sentence.
- It is better to regard the study as comprised of a single, multi-condition experiment, rather than 3 different experiments. ⇒We rephrased “test 1”, “test 2”, and “test 3”, instead of experiment 1, 2, and 3.
- P.7: how were the number of trials per run (before the probability reversals) distributed? How were the reversal points determined?
- Although we misunderstood this question, it was described that in one run, the probability reversals took place in the 31th and 71th trials. For the first 30 trials, the probability remained the same. Then, the probability was reversed, and up to 70^th^ trial, the probability remained the same. The second reversal took place at the 71th trial, and the subsequent trials retained the same probability. Please let us know if this explanation (and that in the article) is still ambiguous.
- Procedure: who was the participant that responded in each trial? For example, did the participants take turns, or was the decision determined by the first participant who answered?
  - In each trial, group members discuss and decide jointly. They communicated via breakout sessions in the Zoom. We described “Group members in tests 2 and 3 freely communicated via Zoom during the session, while sharing test screens in PsytoolKit, and made choices.” in Experiments in Methods.
- what were the specific parameters of the distributions that were used as priors for parameter estimation?
  - We described in Methods: “More specifically, since $\alpha$ ($\alpha^{\pm}$) is bounded between 0 and 1, and $\beta$, take non-negative values, their priors were assumed to follow beta distributions for $\alpha(\alpha^{\pm})$, and gamma distributions for $\beta$.”
- Given the impressive sample-size used in this study, it would be very valuable to provide the readers access to the raw data, as well as the analysis code.
  - Yes, we will upload the raw dataset immediately after the acceptance.
- P. 11 and onward: exact p-values should be given for all statistical tests (significant or not). ⇒ P values were added in all test results in the text.
- P. 12, top: the difference between individuals and dyads does not meet the standard .05 criterion. The same is true for p. 14, top. However, I do not think that the Bonferroni correction is needed when having only 2 comparisons, so that the uncorrected p-value can be used in the former case.
  - This is correct. However, since we were interested in establishing the U-shaped relationship across three groups, we would like to adhere to the multiple comparison framework.
- P. 13, top paragraph: mode details should be given regarding the parameters that did not vary across group sizes (i.e., descriptive and inferential statistics).
- The descriptive statistics were added in the text in Tables 1 and 2.
- A section on parameter recovery is missing. It is important to show the precision in which the parameter values could be recovered using the number of trials and participants used in this study. This can strengthen the claims regarding group-size invariance in some of the parameters.
  - This is important suggestion, but I am not sure whether this parameter recovery is feasible or not, given individual parameter estimations were conducted in this study. My understanding is that the parameter recovery could be applied to a few estimated parameters such as hierarchical Bayesian parameter estimation in which the common parameters are estimated across different individuals. In this study, each individual or each group is assumed to have different parameters. So we estimated parameters for each individual and each group. The total number of parameters amounted to 570 (322 individuals, 138 dyads, and 110 triads). For each sample, 2 parameters in the simple Q model, in which case, the total number of parameters is 1140. So, we are not sure how the parameter recovery for these 1140 estimation results could be reported in the paper.
- Figures 2-4 do not provide much more information than is already given in the text, and hence should be omitted. 14. The bar graphs used to show the data are quite outdated compared to what is acceptable and standard practice these days which includes superimposing the data points on top of the bars and/or showing the distributions using violin plots and similar tools
- These figures were deleted.

Responses to Reviewer 2:

1. Equation 1, phi is never explained. What does it represent and what function does it play in the model? It is also listed on pg. 10 as one of the parameters, but nothing is said about its prior.

- $\phi$ is added here because Q learning tends to generate autocorrelation of the choices, which might bias the estimates of learning parameters, as demonstrated by Katahira (2018). In the text, we added “$\phi$ is added here as the choice trace to account for autocorrelation of choice, which could affect the learning biases (56).”

2. The comments on the previous learning model were correct and we changed the models completely. The two models were much simpler than the previous one, and more standard, used by many related studies. So we hope this time Reviewer 2 did not have any serious concerns about the models.
3. The paper implies that triads do better than individuals, but the difference is not significant, so the authors need to be more careful about how the results are presented. I noticed this in the abstract, but it might say it elsewhere as well

- This is correct and we removed the corresponding sentences. Also in the simple model in Introduction, we showed that Individuals outperform triads if p<.5, but underperform otherwise. Thus, the relationship between the two should and was indeterminate.

4. pg. 8 – the text jumps into explaining the modeling in the section that explains the task. It should probably be its own section

- We created the subsection “Two-armed bandit problem” and explained the game. Then, we moved on to the explanation of the models.

5. top of pg. 4 'put' should be 'puts'
   pg. 5 "single" maybe should be "signal"
   pg. 13. first sentence of 2nd paragraph. 'the' should be 'a'

- We corrected all of these.
