# Supplementary material for: Three heads are better than two: Comparing learning properties and performances across individuals, dyads, and triads through a computational approach
Source: PLoS One. 2021 Jun 17;16(6):e0252122. doi: 10.1371/journal.pone.0252122 (PMC8211165; doi:10.1371/journal.pone.0252122)
Supplement: Attachment — Submitted filename: Revision List.docx [file pone.0252122.s003.docx]

**Revision List**

We greatly appreciate reviewers’ helpful comments. This time, Reviewer 2 seemed to accept the revision. So current revision was made in response to Reviewer 1’ s comments.

Main changes
First, the main revisions in the current manuscript are as follows:

1. The subsample in the previous manuscript was too strict to severely limit the sample size. So this time, we relaxed the criteria of selecting the subsample to compare across different groups. This time, we selected the sample according to the criteria in which at least one member of the dyadic and triadic groups undertook Test 3 and Test 2, respectively. The results remained the same as before, but this time, the size of this subsample was 262 individuals, 116 dyadic groups and 93 triadic groups, much larger than the previous subsample.
2. We described our hypothesis more clearly in Introduction, and moved the algebraic model part to Appendix.
3. In the previous manuscript, we separately examined learning rates of $\alpha^{\pm}$. But this time, we calculated the positivity biases $\alpha^{+}-\alpha^{-}$ because many related studies reported positivity biases emerged for many participants. In this manuscript, it was revealed that positivity biases were found in individuals and triadic groups, but not in dyadic groups. This could be one evidence for learning incoherence in dyadic groups. We also examined this positivity biases in within group effects.
4. The description regarding the number of participants who took tests several times seemed to confuse Reviewer 1. So we rewrote the relevant part to clarify the fact that in each group, some participants undertook tests more than once.

Responses to Reviewer 1:

1. “In the introduction, we see an attempt at motivating a theoretical justification in pp. 6-7. Frankly, I did not understand any of the notation or its relationship to the study or how it could then be a motivation for the experiment.”

- First, we rewrote this part to make it clearer. The hypothesis was as follows:
  - Odd numbered group sizes (individuals and triads) generate higher performance due to learning coherence.
  - Even numbered group sizes (dyads) generate lower performance due to learning incoherence.
  - Learning coherence emerged because majority groups could take initiative in decision making over time. In triadic groups, two members out of three could form majority groups and make decision. In individuals, only one member made decision
  - Learning incoherence was generated due to no such majority subgroups emerged, especially in dyadic groups. In this case, two members agreed or two members did not agree.
  - The simple algebraic model just described this situation and compared expected rewards in individuals, dyads, and triads.
- In the empirical study, first we compared performance across individuals, dyads, and triads. Then, we compared learning properties. We interpreted learning incoherence took place in dyads because the positivity biases did not emerge and the inverse temperature was somewhere between individuals and triads. In triads, they pursue more exploitation strategy. In individuals, they pursue more exploratory strategy. However, dyadic groups took a stuck-in-the-middle strategy, which we interpret as one evidence of learning incoherence. Thus, we believe our hypothesis was supported in the empirical study: Individuals and triads generated higher performance due to learning coherence and dyads generated lower performance due to learning incoherence.
- As for the model part, while it was moved to Appendix, we could also drop this part, if reviewers consider it unnecessary.
- This is more detailed explanation. In the population, we assumed each person adopts either strategy Sh or strategy Sl, each generating the expected rewards of Rh and Rl, respectively (Rh>Rl is assumed). The proportion of Sh is p and that of Sl is 1-p. Then, the expected reward for individuals is

$V1=pRh+\left( 1-p \right)Rl$

We also assumed that the randomized adoption of Sh and Sl generated expected rewards of Rw with Rw<Rl<Rh. Then, in dyads, its expected reward was

$$V2=p^{2}Rh+\left( 1-p \right)^{2}Rl+2p\left( 1-p \right)Rw$$

where $p^{2}$ is the probability that two members adopt Sh, $\left( 1-p \right)^{2}$ is the probability that two members adopt Sl, and $2p\left( 1-p \right)$ is the probability that one member adopt Sh and another Sl. In a similar manner, the expected reward for triads is

$$V3=p^{2}\left( 3-2p \right)Rh+\left( 1-p \right)^{2}\left( 1+2p \right)Rl$$

Taking difference yields

$$V1-V2=pRh+\left( 1-p \right)Rl-p^{2}Rh-\left( 1-p \right)^{2}Rl-2p\left( 1-p \right)Rw$$

$$=p\left( 1-p \right)\left[ Rh+Rl-2Rw \right]>0$$

$$V3-V2=p^{2}\left( 3-2p \right)Rh+\left( 1-p \right)^{2}\left( 1+2p \right)Rl-p^{2}Rh-\left( 1-p \right)^{2}Rl-2p\left( 1-p \right)Rw$$

$$=2p^{2}\left( 1-p \right)Rh+{2p\left( 1-p \right)}^{2}Rl-2p\left( 1-p \right)Rw$$

$$=2p\left( 1-p \right)\left\{ pRh+\left( 1-p \right)Rl-Rw \right\}>0$$

$$V3-V1=p\left( 1-p \right)\left( 2p-1 \right)\left( Rh+Rl \right)$$

Thus, the U-shaped relationship emerged due to learning coherence and

incoherence.

2. “Two RL models are presented, and applied to the data and their fits are equally good and cannot be differentiated. In addition, the models do not make any different predictions for the experiments either. One is left wondering what the purpose of the exercise is.”

- The two RL models were prepared in response to Reviewer 1’s request for the necessity of model comparison. The result was undifferentiated between the two models. We think two responses are possible. One is to drop either model. Since the positivity biases could not be evaluated in the simple model, this model could be dropped. Another possibility is to maintain two models, and examine whether the learning properties remain the same between the two alternative models. We took this alternative and check the robustness of the results. The results indicated that inverse temperatures accounted for higher performance in both models and we believe they show the robustness of the results. Although reporting the results of the two models seemed more persuasive, if Reviewer 1 prefer to drop either model, we will drop the simple model and report the result of the asymmetric model alone.

3. “In the first round, we asked for clarification about which subjects did the 1, 2 or 3-person experiments and how many rounds they did. Here, this request has been addressed but the rebuttal does not really solve any problem. On pp 10-11 (line 172-185) the descriptions are more confusing than helping. For example, in line 184, we are told that 161 individuals participated in triadic experiments but 161 is not divisible by 3. This leaves the reader with the impression that there was no real systematicity to the arrangement of the experimental participation.”

- In this description, we are afraid that some misunderstanding exists. Please note that in the subsample, some individuals undertook tests more than once. Thus, 161 individuals participated in triadic groups, but some individuals participated in these groups more than once. Therefore, the number of individuals (=161) was not necessarily the multiples of three.
- However, taking the fact that our description induced misunderstanding, we rewrote this part with more information. Since we changed the subsample this time, we rewrote the corresponding part.
- Let us examine the relation across the number of individuals and that of dyadic and triadic groups as follows:

Pooled sample

\# of individuals = 322

\# of dyads=138 (230 and 23 individuals took once and twice)

\# of triads=108 (153, 66, 13 individuals took one, two, and three times)

In dyads, the total # of individuals is 276 (=138*2).

230 individuals once

\# of individuals taking twice = 46 (=23*2)

Sum: 230+46=276

In triads, the total # of individuals is 324 (=108*3)

153 individuals once

\# of individuals taking twice = 132 (=66*2)

\# of individuals taking three times = 39 (=13*3)

Sum: 153+132+39=324

Note that some participants undertook both Tests 2 and 3. We described this information regarding the subsample because it was relevant to group comparison, which used the subsample alone in this manuscript.

- In the subsample,

\# of individuals = 262

\# of dyads=116 (200 and 16 individuals took once and twice)

\# of triads=93 (142, 52, 11 individuals took one, two, and three times)

In dyads, the total # of individuals is 232 (=116*2).

200 individuals taking once

\# of individuals taking twice = 32 (=16*2)

Sum: 200+32=232

In triads, the total # of individuals is 279 (=93*3)

142 individuals taking once

\# of individuals taking twice = 104 (=52*2)

\# of individuals taking three times = 33 (=11*3)

Sum: 142+104+33=279

We also provided the information for those taking either or both Tests 2 and 3 in the subsample because this was related to group comparison.

The total # of individuals taking Tests 2 and 3=511(=116 dyads*2+93 triads*3)

\# of individuals taking these tests once = 84

\# of individuals taking these tests twice=118

\# of individuals taking these test three times = 49

\# of individuals taking these test four times=11

Sum: Total # of individuals = 84+118*2+49*3+11*4=511

\# of individuals = 84+118+49+11=262

- The related information was provided in the following sentences:

“All participants in this sample undertook Test 1 once and at least one more experiment in Tests 2 or 3. In Test 2 (dyads), 230 and 23 participants played the TAB once and twice, respectively. In Test 3 (triads), 153, 66 and 13 participants played one, two, and three times, respectively.”

” In total, 262 individuals were in this subsample. In this subsample, 116 dyadic groups and 93 triadic groups were identified. In Tests 2 and 3, the numbers of individuals who took the tests one, two, three, and four times were 84, 118, 49, and 11, respectively. Of the dyads, 216 individuals participated, and 200 and 16 undertook Test 2 once and twice, respectively. Of the triads, 205 individuals participated, and 142, 52 and 11 individuals undertook Test 3 one, two and three times, respectively.”
